# Fractional Deng Entropy and Extropy and Some Applications

**DOI:** 10.3390/e23050623

**Published:** 2021-05-17

**Authors:** Mohammad Reza Kazemi, Saeid Tahmasebi, Francesco Buono, Maria Longobardi

**Affiliations:** 1 Department of Statistics, Faculty of Science, Fasa University, Fasa 746-168-6688, Iran; kazemi@fasau.ac.ir; 2 Department of Statistics, Persian Gulf University, Bushehr 751-691-3817, Iran; tahmasebi@pgu.ac.ir; 3 Dipartimento di Matematica e Applicazioni “Renato Caccioppoli”, Università degli Studi di Napoli Federico II, 80138 Naples, Italy; francesco.buono3@unina.it; 4 Dipartimento di Biologia, Università degli Studi di Napoli Federico II, 80138 Naples, Italy

**Keywords:** measures of uncertainty, fractional entropy, Deng entropy and extropy, classification and discrimination, 62H30, 62B10, 94A17

## Abstract

Deng entropy and extropy are two measures useful in the Dempster–Shafer evidence theory (DST) to study uncertainty, following the idea that extropy is the dual concept of entropy. In this paper, we present their fractional versions named fractional Deng entropy and extropy and compare them to other measures in the framework of DST. Here, we study the maximum for both of them and give several examples. Finally, we analyze a problem of classification in pattern recognition in order to highlight the importance of these new measures.

## 1. Introduction

The concept of entropy as a measure of uncertainty was first introduced by Shannon [1], and since then, it has been used in the field of information theory, image and signal processing and economics. Let *X* be a discrete random variable with probability mass function vector $\underline{p}=\left(p_{1},\ldots ,p_{n}\right)$. The Shannon entropy of *X* is defined as follows

(1) $$H\left(X\right)=H\left(\underline{p}\right)=-\sum_{i=1}^{n}p_{i}\log p_{i},$$

where log(·) stands for the natural logarithm with the convention 0log0=0. Recently, the dual measure of entropy has become widespread. It is known as extropy and was defined for a discrete random variable *X* by Lad et al. [2] as

(2) $$J\left(X\right)=J\left(\underline{p}\right)=-\sum_{i=1}^{n}\left(1-p_{i}\right)\log\left(1-p_{i}\right),$$

and since then, as the Shannon entropy, it has been studied in several contexts and in its differential version [3,4,5,6].

The generalization of Shannon entropy to various fields is always of great interest. Ubriaco [7] defined a new entropy based on fractional calculus as follows:

(3) $$S_{q}\left(X\right)=S_{q}\left(\underline{p}\right)=\sum_{i=1}^{n}p_{i}{\left[-\log p_{i}\right]}^{q},\;\;0<q\leq 1.$$

The fractional entropy is concave, positive and non-additive. Moreover, for q=1, the fractional entropy reduces to the Shannon entropy. From a physical sense, it also satisfies Lesche and thermodynamic stability.

The purpose of this paper is to extend to the fractional case of Deng entropy and extropy. Deng entropy and extropy [8,9] are two measures of uncertainty known in the context of the Dempster–Shafer theory (DST) of evidence. The DST of evidence [10,11] is a generalization of the classical probability theory. In DST, an uncertain event with a finite number of alternatives is considered, and a mass function over the power set of the alternatives, considered as a degree of confidence, is defined. DST allows us to describe more general situations in which there is less specific information with respect to the classical probability theory. DST has several applications due to its advantages in dealing with uncertainty; for example, it is used in reliability analysis [12,13], in decision making [14,15], and so on [16,17].

Now, we describe an example given in [8] to explain how DST extends the classical probability theory. Consider two boxes, *A* and *B*, such that in *A*, there are only red balls, whereas in *B*, there are only green balls and the number of balls in each box is unknown. A ball is picked randomly from one of the boxes. The box *A* is chosen with probability $p_{A}=0.6$ and box *B* is selected with probability $p_{B}=0.4$. Thus, the probability of picking up a red ball is 0.6, P(R)=0.6, and the probability of picking a green ball is 0.4, P(G)=0.4. Now, suppose in box *B* there are green and red balls with rates unknown and $p_{A}$, $p_{B}$ are unchanged. In this case, we cannot obtain the probability of picking up a red ball. To overcome this problem, we can use DST to express the uncertainty. In particular, we choose a mass function *m*, such that m(R)=0.6 and m(R,G)=0.4.

The rest of the paper is organized as follows. In Section 2, we recall the basic notions of the Dempster–Shafer theory of evidence and some of the most important measures of uncertainty in this context. In Section 3, we define and study the fractional Deng entropy. In Section 4, we introduce the fractional Deng extropy, and several examples are given. In Section 5, we apply fractional Deng entropy and fractional Deng extropy to a problem of classification. Finally, in Section 6, we give conclusions and summarize the results obtained in the paper.

## 2. Preliminaries

In this section, we review some basic definitions in the Dempster–Shafer evidence theory (DST) [10,11] and Deng entropy [8].

**Definition** **1.** *Let $X=\{\theta_{1},\theta_{2},\cdots ,\theta_{i},\cdots ,\theta_{\left|X\right|}\}$ be a finite set of mutually exclusive and collectively exhaustive events, X is the frame of discernment (FOD). The power set of X consists of $2^{|X|}$ elements denoted as follows:*

$$2^{X}=\left\{\emptyset ,\left\{\theta_{1}\right\},\cdots ,\left\{\theta_{\left|X\right|}\right\},\left\{\theta_{1},\theta_{2}\right\},\cdots ,\left\{\theta_{1},\theta_{2},\cdots ,\theta_{i}\right\},\cdots ,X\right\}.$$

**Definition** **2.** *(Mass function) Given a FOD $X=\{\theta_{1},\theta_{2},\cdots ,\theta_{i},\cdots ,\theta_{\left|X\right|}\}$, a mapping m from $2^{X}$ to [0,1] is called a mass function, or basic probability assignment (BPA), formally defined by:*

$$m:\;\;2^{X}\to \left[0,1\right]$$

*which satisfies*

(4) $$m\left(\emptyset \right)=0,\;\;\sum_{A\in 2^{X}}^{}m\left(A\right)=1,\;\;m\left(A\right)\geq 0.$$

*In DST, m(A) represents how strongly the evidence supports A. Then, m(A) measures the belief exactly assigned to A. If m(A)>0, then A is called a focal element.*

Recently, some operations on BPA are presented, such as negation [18] and correlation [19]. In several applications, we need to generate a new BPA starting from independent BPAs or from a weight of evidence represented by a coefficient α∈(0,1].

In DST, there are different indices to evaluate the degree of belief in a subset of FOD. Among them, here we recall the definitions of belief function, plausibility function and pignistic probability transformation (PPT).

**Definition** **3.** *(Belief function and plausibility function) A BPA m can also be represented by the belief function Bel or the plausibility function Pl, defined as follows:*

$$\begin{array}{c} Bel\left(A\right)=\sum_{\emptyset \neq B\subseteq A}^{}m\left(B\right),\;\;Pl\left(A\right)=\sum_{B\cap A\neq \emptyset }^{}m\left(B\right). \end{array}$$

**Definition** **4.** *Given a BPA m on a FOD X, the pignistic probability transformation (PPT) of A⊆X is defined as [20]*

(5) $$PPT\left(A\right)=\sum_{B:A\subseteq B}\frac{m(B)}{\left|B\right|}.$$

### Some Uncertainty Measures for the Dempster–Shafer Framework

In the context of the DST, there are interesting measures of discrimination, such as Deng entropy; it has many advantages in some cases, in comparison with other uncertainty measures in the DST framework. It was this latter concept that has suggested to us the introduction of a new extension. In Table 1, we present the definitions of some of the most important measures of uncertainty in DST.

**Definition** **5.** *(Deng entropy) Deng entropy was introduced in [8] for a BPA m as*

(6) $$\begin{array}{c} E_{d}\left(m\right)=-\sum_{A\subseteq X:m(A)>0}^{}m\left(A\right)\log_{2}\left(\frac{m(A)}{2^{\left|A\right|}-1}\right), \end{array}$$

*where |A| denotes the cardinality of the focal element A.*

Deng entropy degenerates to the Shannon entropy if, and only if, a positive mass function value is assigned only to singleton elements, which is $E_{d}\left(m\right)=-\sum_{i=1}^{|X|}m\left(\left\{\theta_{i}\right\}\right)\log_{2}m\left(\left\{\theta_{i}\right\}\right).$ Deng entropy has attracted the interest of researchers, and several of its generalizations have been studied. In Table 2, we present some modified versions of Deng entropy.

## 3. Fractional Deng Entropy

In recent years, great attention has been given to fractional calculus. For this reason, several authors have studied various fractional entropies from the idea that they satisfy physical conditions of stability. In order to obtain an analog of (6), we introduce the concept of fractional Deng entropy in the following definition.

**Definition** **6.** *Let m be a BPA on a FOD X. We define the Fractional Deng Entropy (FDEn) of m as*

(7) $$E_{d}^{q}\left(m\right)=\sum_{A\subseteq X:m(A)>0}^{}m\left(A\right){\left[-\log_{2}\left(\frac{m(A)}{2^{\left|A\right|}-1}\right)\right]}^{q},\;\;0<q\leq 1.$$

**Example** **1.**

*(i)*  *Assume that the FOD is X={a,b,c}. For a mass function $m\left(a\right)=m\left(b\right)=m\left(c\right)=\frac{1}{3}$, the associated fractional entropy and FDEn are obtained as follows:*
  $$S_{q}\left(\underline{p}\right)=E_{d}^{q}\left(m\right)={\left[\log_{2}3\right]}^{q}.$$
  *It is obvious that, in this case, the FDEn is increasing in q∈(0,1].*
*(ii)*  *Assume there is a∈X such that m(a)=1. The associated fractional entropy and FDEn coincide and are obtained as*
  $$S_{q}\left(\underline{p}\right)=E_{d}^{q}\left(m\right)=0.$$

Clearly, we see that the results of fractional entropy and FDEn are identical when the BPA assigns a positive mass only to singletons. Moreover, if A⊆X exists such that m(A)>0 and ∣A∣>1, we cannot evaluate the fractional entropy.

**Example** **2.** *Given a FOD X={a,b,c}, for a mass function $m_{1}\left(a,b,c\right)=1$, we have*

$$E_{d}^{q}\left(m_{1}\right)={\left[\log_{2}7\right]}^{q}.$$

*For another mass function $m_{2}\left(a\right)=m_{2}\left(b\right)=m_{2}\left(c\right)=m_{2}\left(a,b\right)=m_{2}\left(a,c\right)=m_{2}\left(b,c\right)=m_{2}\left(a,b,c\right)=\frac{1}{7}$, we obtain*

$$E_{d}^{q}\left(m_{2}\right)=\frac{3}{7}\left({\left[\log_{2}7\right]}^{q}+{\left[\log_{2}21\right]}^{q}\right)+\frac{1}{7}{\left[\log_{2}49\right]}^{q}.$$

**Figure 1:**
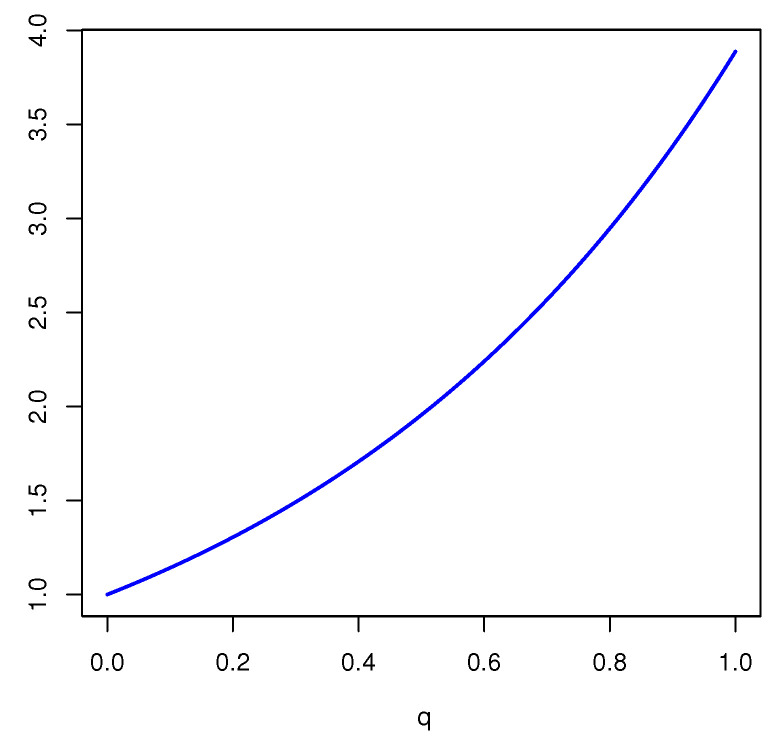
Plot of $E_{d}^{q}\left(m_{2}\right)$ in Example 2 as a function of *q*.

*The plot of the FDEn as a function of q∈(0,1] is given in Figure 1. From Figure 1, it is seen that $E_{d}^{q}\left(m\right)$ is increasing in q and the maximum is achieved for q=1, i.e., when the FDEn reduces to Deng entropy.*

**Example** **3.** *Assume that the FOD is $X=\{a_{1},a_{2},\cdots ,a_{20}\}$. For a mass function $m(\left\{a_{1},a_{2},\cdots ,a_{10}\right\})=0.4$, $m(\left\{a_{11},a_{12},\cdots ,a_{20}\right\})=0.6,$ we obtain*

$$E_{d}^{q}\left(m\right)=0.4{\left[-\log_{2}\left(\frac{0.4}{2^{10}-1}\right)\right]}^{q}+0.6{\left[-\log_{2}\left(\frac{0.6}{2^{10}-1}\right)\right]}^{q}.$$

**Figure 2:**
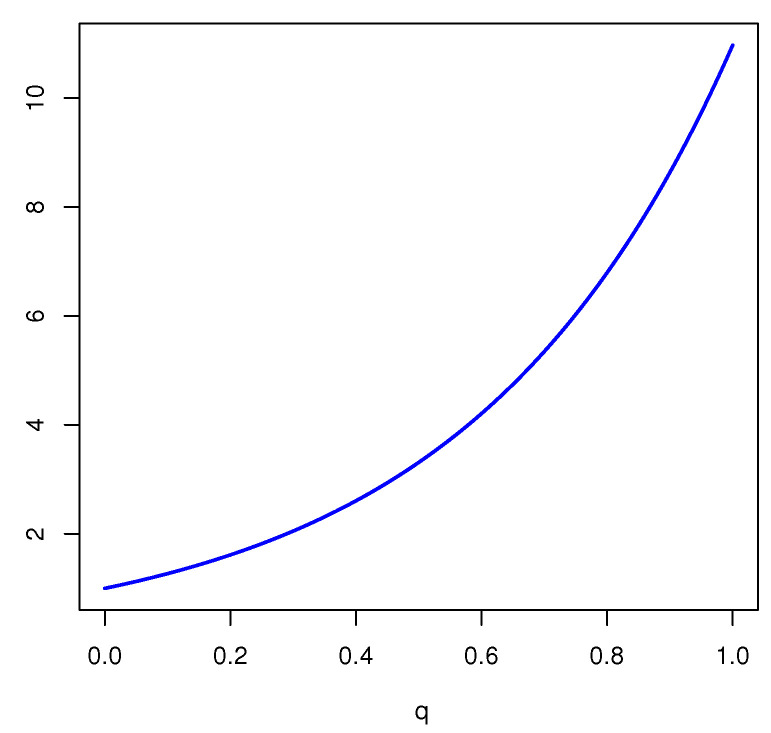
Plot of $E_{d}^{q}\left(m\right)$ in Example 3 as a function of *q*.

*The plot of this FDEn is given in Figure 2. From Figure 2, it is seen that $E_{d}^{q}\left(m\right)$ is increasing in q, and the maximum is achieved when FDEn reduces to Deng entropy.*

**Example** **4.** *Let us consider a FOD X={a,b,c} and a BPA m such that $m\left(a\right)=p_{a}$ and $m\left(a,b\right)=r_{a}$, where $r_{a}=1-p_{a}$. For $p_{a}\in \left\{0.01,0.8,0.99\right\}$, the function $E_{d}^{q}\left(m\right)$ is computed. In this example, it is shown that the $E_{d}^{q}\left(m\right)$ can be increasing, decreasing and upside-down bath-tubed shaped. The FDEn is given by*

$$E_{d}^{q}\left(m\right)=p_{a}{\left[\log_{2}\left(1/p_{a}\right)\right]}^{q}+r_{a}{\left[\log_{2}\left(3/r_{a}\right)\right]}^{q}.$$

**Figure 3:**
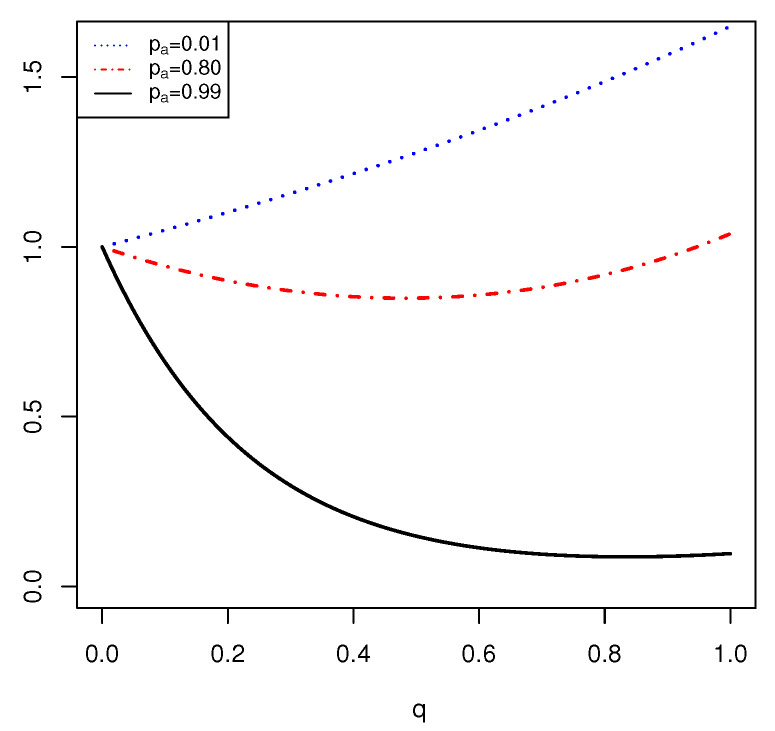
Plot of $E_{d}^{q}\left(m\right)$ in Example 4 as a function of q for different values of $p_{a}$.

*In Figure 3, the plot of $E_{d}^{q}\left(m\right)$ for different values of $p_{a}$ is given. It is seen that for $p_{a}=0.01$, $p_{a}=0.80$ and $p_{a}=0.99$, the plot of $E_{d}^{q}\left(m\right)$ is increasing, upside-down bathtub shaped and decreasing, respectively.*

In the above examples, it is seen that the function $E_{d}^{q}\left(m\right)$ cannot be a concave function, and it can be increasing, decreasing and upside-down bathtub shape. Furthermore, the supremum FDEn is achieved when *q* is near to the boundary of interval (0,1]. Therefore, we can state the following theorem.

**Theorem** **1.** *Let m be a non-degenerate BPA on a FOD X and q∈(0,1]. Then, the supremum FDEn as a function of q is attained for q∈{0,1} and the infimum is attained in the extremes of interval (0,1), or it is a minimum assumed in a unique $q_{0}\in \left(0,1\right)$.*

**Proof.**  By noting that for fixed x>0 the function $g\left(p\right)=x^{p}$ is a convex function of *p* we can conclude that the FDEn is a strictly convex function of *q*. Hence, we have three possible scenarios. In the first one, the FDEn is strictly increasing in *q* and hence it assumes the maximum value for q=1, i.e., when it reduces to Deng entropy, and the infimum is 1 by the normalization condition. In the second scenario, the FDEn is strictly decreasing; hence, the supremum is 1 and the minimum is assumed for q=1. In the third case, there is a unique stationary point in (0,1), it is an absolute minimum, whereas the supremum is given by $\max\{1,E_{d}\left(m\right)\}$. □

In the following theorem, we study the maximum FDEn for a fixed value of *q*. This is an important issue in the theory of measures of uncertainty; see, for instance, [30] for the study of the maximum Deng entropy.

**Theorem** **2.** *Let X be a FOD, q∈(0,1] and m be a BPA, which assigns positive mass to each non-empty subset of X. The maximum FDEn is attained if the BPA m is defined as*

(8) $$m\left(A\right)=\frac{2^{\left|A\right|}-1}{\sum_{B\subseteq X}\left(2^{\left|B\right|}-1\right)},\;\;A\subseteq X.$$

**Proof.**  For a fixed q∈(0,1] the FDEn is given by (7) as

(9) $$E_{d}^{q}\left(m\right)=\sum_{\emptyset \neq A\subseteq X}m\left(A\right){\left[-\log_{2}\left(\frac{m(A)}{2^{\left|A\right|}-1}\right)\right]}^{q}.$$

We have to maximize (9) subject to the constraint

(10) $$\sum_{\emptyset \neq A\subseteq X}m\left(A\right)=1.$$

We use the method of Lagrange multipliers, and we have to compute the partial derivatives of the function

$${\tilde{E}}_{d}^{q}=\sum_{\emptyset \neq A\subseteq X}m\left(A\right){\left[-\log_{2}\left(\frac{m(A)}{2^{\left|A\right|}-1}\right)\right]}^{q}+\lambda \left(\sum_{\emptyset \neq A\subseteq X}m\left(A\right)-1\right)$$

with respect to m(A). By differentiating ${\tilde{E}}_{d}^{q}$ with respect to m(A), we have

$$\begin{array}{ccc} \frac{\partial {\tilde{E}}_{d}^{q}}{\partial m(A)} & = & {\left[-\log_{2}\left(\frac{m(A)}{2^{\left|A\right|}-1}\right)\right]}^{q}-q\log_{2}\left(e\right){\left[-\log_{2}\left(\frac{m(A)}{2^{\left|A\right|}-1}\right)\right]}^{q-1}+\lambda \\ & = & {\left[-\log_{2}\left(\frac{m(A)}{2^{\left|A\right|}-1}\right)\right]}^{q-1}\left[-q\log_{2}\left(e\right)-\log_{2}\left(\frac{m(A)}{2^{\left|A\right|}-1}\right)\right]+\lambda . \end{array}$$

In order to vanish all the partial derivatives of ${\tilde{E}}_{d}^{q}$, the ratio $\frac{m(A)}{2^{\left|A\right|}-1}=K$ has to be invariant with respect to *A*. In fact, the function

$$g\left(z\right)={\left[-\log_{2}\left(z\right)\right]}^{q-1}\left[-q\log_{2}\left(e\right)-\log_{2}\left(z\right)\right]$$

is strictly decreasing in z∈(0,1) since

$$g^{\prime }\left(z\right)=\frac{q\log_{2}\left(e\right)}{z}{\left[-\log_{2}\left(z\right)\right]}^{q-2}\left[\left(q-1\right)\log_{2}\left(e\right)+\log_{2}\left(z\right)\right]$$

and $z<e^{1-q}$. Hence, by the constraint (10), we get

$$K=\frac{1}{\sum_{B\subseteq X}\left(2^{\left|B\right|}-1\right)}$$

and the BPA *m*, which maximizes the FDEn, is given in (8). □

**Example** **5.** *Based on the result of Theorem 2, let us evaluate the maximum FDEn for a FOD of cardinality 3, X={a,b,c}. In this case, the BPA given in (8) is defined as*

$$\begin{array}{cc} \; & m\left(a\right)=m\left(b\right)=m\left(c\right)=\frac{1}{19},\\ \; & m\left(a,b\right)=m\left(a,c\right)=m\left(b,c\right)=\frac{3}{19},\\ \; & m\left(X\right)=\frac{7}{19}. \end{array}$$

*Then, the maximum FDEn is given by*

$$E_{d}^{q}\left(m\right)={\left[\log_{2}\left(19\right)\right]}^{q}.$$

## 4. Fractional Deng Extropy

In the following definition, we present the Deng extropy introduced by Buono and Longobardi [9] as a dual measure of uncertainty to Deng entropy.

**Definition** **7.** *(Deng Extropy) Deng extropy was introduced in [9] for a BPA m on a FOD X as*

$$\begin{array}{c} EX_{d}\left(m\right)=-\sum_{A\subset X:m(A)>0}^{}\left(1-m\left(A\right)\right)\log_{2}\left(\frac{1-m(A)}{2^{|A^{c}|}-1}\right), \end{array}$$

*where $A^{c}$ is the complementary of A in X and $|A^{c}\left|=|X|-|A\right|$.*

Now, in analogy with FDEn, we introduce the fractional version of Deng extropy.

**Definition** **8.** *Let m be a BPA on a FOD X. We define the Fractional Deng Extropy (FDEx) of m as*

(11) $$\begin{array}{c} EX_{d}^{q}\left(m\right)=\sum_{A\subset X:m(A)>0}^{}\left(1-m\left(A\right)\right){\left[-\log_{2}\left(\frac{1-m(A)}{2^{|A^{c}|}-1}\right)\right]}^{q}. \end{array}$$

**Example** **6.**

*(i)*  *Assume that the FOD is X={a,b,c}. For a mass function $m\left(a\right)=m\left(b\right)=m\left(c\right)=\frac{1}{3}$, the associated FDEx is obtained as follows:*
  $$\;EX_{d}^{q}\left(m\right)=2{\left[\log_{2}\frac{9}{2}\right]}^{q}.$$

**Figure 4:**
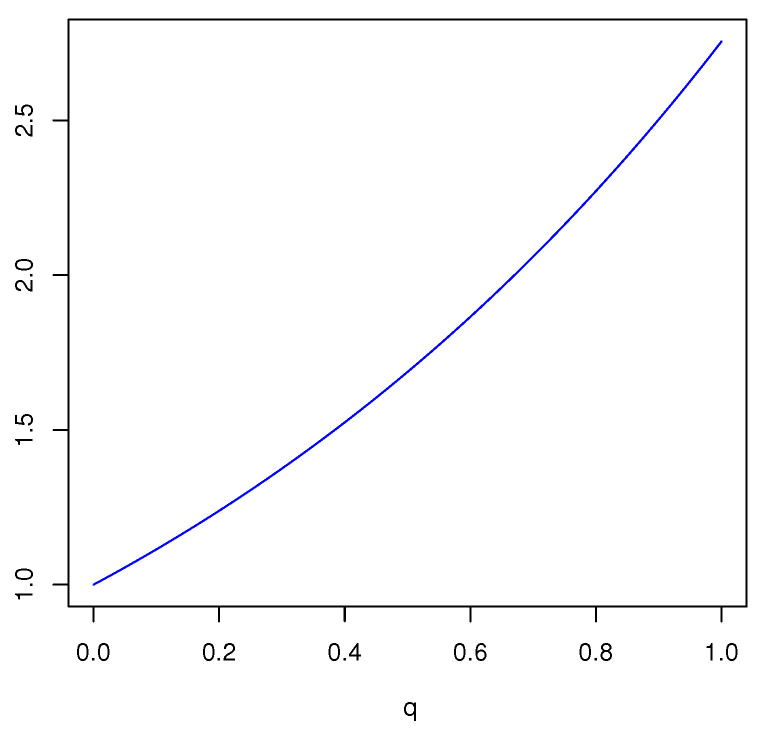
Plot of $EX_{d}^{q}\left(m\right)-E_{d}^{q}\left(m\right)$ in Example 6 as a function of *q*.

*Based on this BPA, we have obtained the FDEn in Example 1. In Figure 4, the plot of $EX_{d}^{q}\left(m\right)-E_{d}^{q}\left(m\right)$ is given.*

*One can see that $EX_{d}^{q}\left(m\right)-E_{d}^{q}\left(m\right)$ is an increasing function of q, and this function is greater than 1. Thus, for q∈(0,1], the FDEx is greater than the FDEn. Furthermore, $EX_{d}^{q}\left(m\right)$ is increasing in q and the maximum is achieved for q=1, i.e., when FDEx reduces to Deng extropy.*

*(ii)*  *Assume there is a∈X such that m(a)=1. Then,*
  $$\;EX_{d}^{q}\left(m\right)=0.$$

*In this case, the FDEx is consistent with its dual definition FDEn.*

**Example** **7.** *Let us consider a FOD X={a,b,c}. For a mass function $m\left(a\right)=m\left(b\right)=m\left(c\right)=m\left(a,b\right)=m\left(a,c\right)=m\left(b,c\right)=m\left(a,b,c\right)=\frac{1}{7}$, we obtain*

$$E_{d}^{q}\left(m\right)=\frac{18}{7}\left({\left[\log_{2}7-1\right]}^{q}+{\left[\log_{2}7-\log_{2}6\right]}^{q}\right).$$

**Figure 5:**
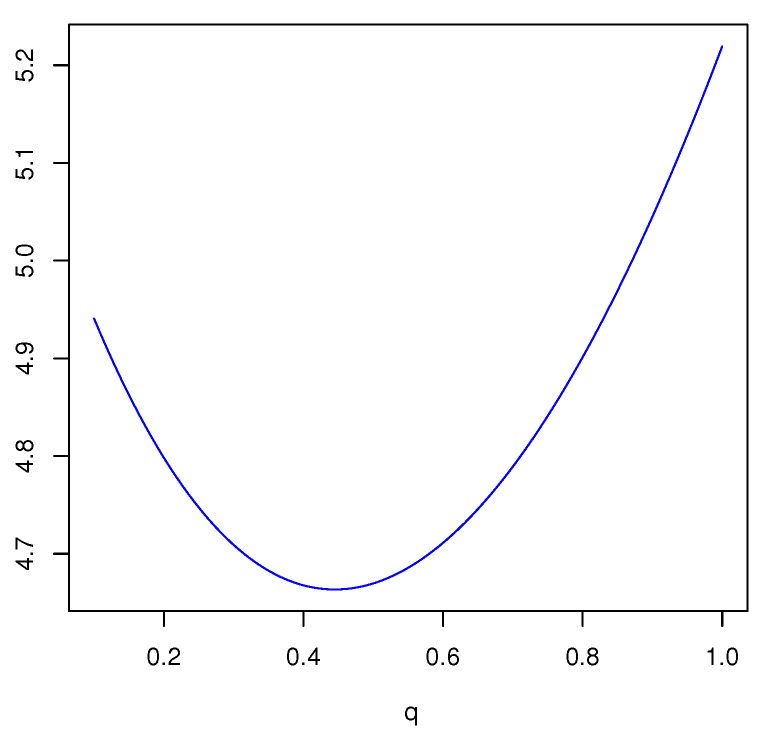
Plot of $EX_{d}^{q}\left(m\right)$ in Example 7 as a function of *q*.

*In Figure 5, the plot of $EX_{d}^{q}\left(m\right)$ is given. One can see that as a function of q, it has a convex parabolic shape and the maximum is achieved when it reduces to Deng extropy.*

**Example** **8.** *Assume that the FOD is $X=\{a_{1},a_{2},\ldots ,a_{20}\}$. For a mass function $m(\left\{a_{1},a_{2},\ldots ,a_{10}\right\})=0.4,$$m(\left\{a_{10},a_{11},\ldots ,a_{20}\right\})=0.6,$ we obtain*

$$EX_{d}^{q}\left(m\right)=0.6{\left[-\log_{2}\left(\frac{0.6}{2^{10}-1}\right)\right]}^{q}+0.4{\left[-\log_{2}\left(\frac{0.4}{2^{10}-1}\right)\right]}^{q}.$$

*In this case, FDEx and FDEn are equal.*

**Example** **9.** *Given a FOD X={a,b,c} and a BPA m such that m(a)=0.9, m(a,b)=0.01 and m(X)=0.09, we have*

$$EX_{d}^{q}\left(m\right)=0.1{\left[\log_{2}30\right]}^{q}+0.99{\left[\log_{2}\frac{100}{99}\right]}^{q}.$$

**Figure 6:**
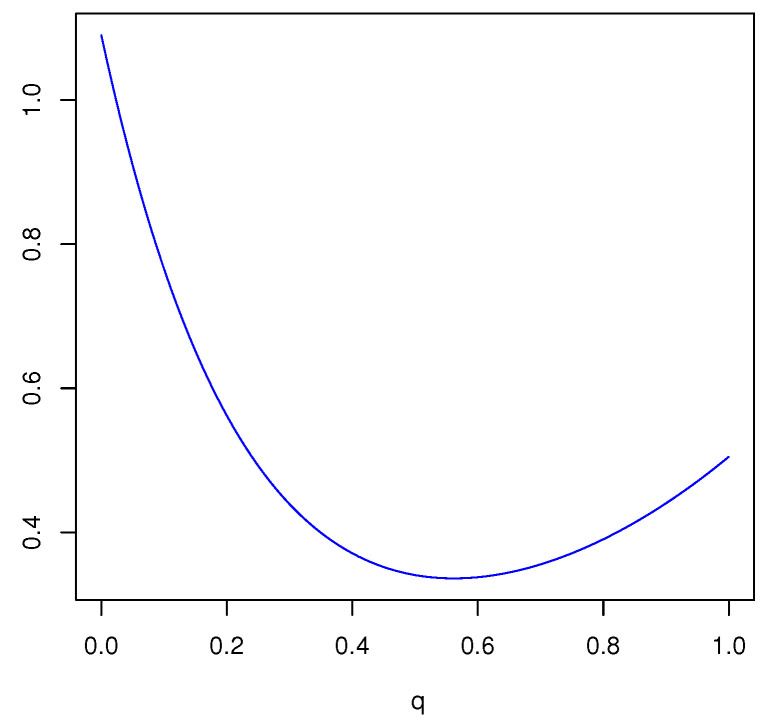
Plot of $EX_{d}^{q}\left(m\right)$ in Example 9 as a function of *q*.

*In Figure 6, the plot of $EX_{d}^{q}\left(m\right)$ is given. One can see that as a function of q, it has a convex parabolic shape and the maximum is achieved when q tends to zero.*

Similar to FDEn, in the above examples, it is seen that the function $EX_{d}^{q}\left(m\right)$ cannot be a concave and it can be increasing, decreasing and upside-down bathtub shape. Furthermore, the supremum FDEx is achieved when *q* is near the boundary of interval (0,1]. The following theorem is immediate.

**Theorem** **3.** *Let m be a non-degenerate BPA on a FOD X and q∈(0,1]. Then, the supremum FDEx as a function of q is attained for q∈{0,1} and the infimum is attained in the extremes of interval (0,1) or it is a minimum assumed in a unique $q_{0}\in \left(0,1\right)$.*

**Proof.**  The proof is similar to that of Theorem 1; in this case, the supremum is given by $\max\{N-1+m\left(X\right),EX_{d}\left(m\right)\}$, where *N* is the number of focal elements different form *X*. □

Next, in analogy with Theorem 2, we obtain an upper bound for the maximum FDEx with a fixed value of *q*.

**Theorem** **4.** *Let X be a FOD, q∈(0,1] and m be a BPA that assigns positive mass to each non-empty subset of X. For a fixed value of m(X), an upper bound for the FDEx is assumed in correspondence of the fictitious BPA $\tilde{m}$ such that $\tilde{m}\left(X\right)=m\left(X\right)$ and*

(12) $$\tilde{m}\left(A\right)=1-\frac{2^{\left|X\right|}-3+m\left(X\right)}{\sum_{\emptyset \neq B\subset X}\left(2^{|B^{c}|}-1\right)}\left(2^{|A^{c}|}-1\right),\;\;\emptyset \neq A\subset X.$$

**Proof.**  The proof is similar to the one given for Theorem 2. After establishing that $\frac{1-m(A)}{2^{|A^{c}|}-1}=K$ have to be invariant with respect to *A*, in order to satisfy the condition of normalization, we get

$$1-m\left(A\right)=K\left(2^{|A^{c}|}-1\right)$$

and, by summing over A⊂X

$$K=\frac{2^{\left|X\right|}-3+m\left(X\right)}{\sum_{\emptyset \neq A\subset X}\left(2^{|A^{c}|}-1\right)}.$$

Hence, the BPA which maximizes the FDEx is given in (12). We have to specify that it is a fictitious BPA, in the sense that $\tilde{m}\left(A\right)$ may be negative for some subset of *X*. □

**Example** **10.** *Based on the result of Theorem 4, let us evaluate the upper bound for FDEx in the case |X|=3 with fixed m(X). We have three subsets of cardinality one and three of cardinality two, and then the upper bound in given by*

$$\begin{array}{ccc} U & = & 3\cdot \frac{3(5+m(X))}{12}{\left[-\log_{2}\left(\frac{5+m(X)}{12}\right)\right]}^{q}+3\cdot \frac{5+m(X)}{12}{\left[-\log_{2}\left(\frac{5+m(X)}{12}\right)\right]}^{q}\\ & = & \left(5+m\left(X\right)\right){\left[\log_{2}\left(\frac{12}{5+m(X)}\right)\right]}^{q}. \end{array}$$

## 5. Application to a Problem of Classification

In this section, we apply FDEn and FDEx to a problem of classification. We analyze a dataset given in [31] about typical qualities of Italian wines. This dataset is composed of 178 instances and, for each one, thirteen attributes are given. The instances of the dataset are divided into three classes of wine: class 1, class 2 and class 3. We use six attributes to discriminate for each instance the correct class. In particular, the attributes involved in this example are: Alcohol, Malic acid, Ash, OD280/OD315 of diluted wines (OD), Color intensity (CI) and Proline. We use the method of max–min values to generate a model of interval numbers. In particular, for a fixed attribute, we study the interval of variability in a single class, and then we intersect the intervals of more classes. The model of interval numbers is shown in Table 3.

Suppose the selected instance is (13.860,1.5100,2.6700,3.1600,3.3800,410). From the dataset, we know that the selected instance belongs to class 2, and our purpose is to classify it in the right way. We generate six BPAs, one for each attribute, by using a method based on the similarity of interval numbers proposed by Kang et al. [32]. Given two intervals $A=[a_{1},a_{2}]$ and $B=[b_{1},b_{2}]$, their similarity S(A,B) can be defined as

$$S\left(A,B\right)=\frac{1}{1+\alpha \;D(A,B)},$$

where α>0 is the coefficient of support, here we use α=5, and D(A,B) is the distance of intervals *A* and *B* defined in [33] as

$$D^{2}\left(A,B\right)={\left[\left(\frac{a_{1}+a_{2}}{2}\right)-\left(\frac{b_{1}+b_{2}}{2}\right)\right]}^{2}+\frac{1}{3}\left[{\left(\frac{a_{2}-a_{1}}{2}\right)}^{2}+{\left(\frac{b_{2}-b_{1}}{2}\right)}^{2}\right].$$

For each attribute, we can get seven values of similarity by choosing as *A* the intervals given in Table 3 and as *B* the corresponding singleton of the selected instance. Then, by normalizing the obtained values, we get six BPAs, as reported in Table 4.

Without any additional information, we can evaluate a final BPA giving the same weight to each attribute, i.e., by summing the six values related to a focal element and then dividing by six. In this way, we get the final BPA shown in Table 5.

Now, based on the BPA in Table 5, we can evaluate the PPT (5) of the classes, and we get

PPT(1)=0.3500,PPT(2)=0.3464,PPT(3)=0.3036.

Hence, the focal element with the highest PPT is class 1, and so, it would be our final hypothesis without making the correct decision.

We try to improve the described method by using FDEn. Let us fix the value q=0.6. We evaluate the FDEn of BPAs given in Table 4 and we obtain the results shown in Table 6.

Since a higher value of FDEn means a higher uncertainty, we can give more weight to the attributes with lower FDEn. In particular, we define the weights by normalizing to 1 the reciprocal values of fractional Deng entropies. We obtain the weights presented in Table 7.

Based on the weights in Table 7, we get a weighted version of the final BPA, as shown in Table 8.

Finally, based on the BPA in Table 8, we evaluate the PPT of the classes and we get

PPT(1)=0.3426,PPT(2)=0.3499,PPT(3)=0.3075.

Hence, the focal element with the highest PPT is class 2, so it is our final hypothesis and we made the correct decision.

Along the same lines, we can use FDEx. In Table 9, we give the recognition rates of the non-weighted method and methods based on FDEn and FDEx for different choices of *q*.

## 6. Conclusions

In this paper, fractional Deng entropy and extropy have been defined from the definitions of Deng entropy and extropy. These measures have been compared with other well-known ones, and some examples have been proposed. Characterization results for the maximum fractional Deng entropy and extropy have been given, and finally, a problem of classification based on a dataset has been discussed in order to emphasize the relevance of these measures in pattern recognition.

## Figures and Tables

**Table 1:** Uncertainty measures in the DST framework.

Uncertainty Measure	Definition
Hohle’s confusion measure [21]	$C_{H}\left(m\right)=-\sum_{A\subseteq X}^{}m\left(A\right)\log_{2}Bel\left(A\right)$
Yager’s Dissonance Measure [22]	$E_{Y}\left(m\right)=-\sum_{A\subseteq X}^{}m\left(A\right)\log_{2}Pl\left(A\right)$
Dubois and Prade’sWeighted Hartley Entropy [23]	$E_{DP}\left(m\right)=-\sum_{A\subseteq X}^{}m\left(A\right)\log_{2}\left|A\right|$
Klir and Ramer’s discord measure [24]	$D_{KR}\left(m\right)=-\sum_{A\subseteq X}^{}m\left(A\right)\log_{2}\sum_{B\subseteq X}^{}m\left(B\right)\frac{\left|A\cap B\right|}{\left|B\right|}$
Klir and Parviz’s strife measure [25]	$S_{KP}\left(m\right)=-\sum_{A\subseteq X}m\left(A\right)\log_{2}\sum_{B\subseteq X}^{}m\left(B\right)\frac{\left|A\cap B\right|}{\left|A\right|}$
George and Pal’s total conflict measure [26]	$TC_{GP}\left(m\right)=\sum_{A\subseteq X}m\left(A\right)\sum_{B\subseteq X}m\left(B\right)\left(1-\frac{\left|A\cap B\right|}{\left|A\cup B\right|}\right)$

**Table 2:** Modified Deng entropy in the DST framework.

Uncertainty Measure	Definition
Zhou et al.’s Entropy [27]	$E_{Md}\left(m\right)=-\sum_{A\subseteq X}^{}m\left(A\right)\log_{2}\left(\frac{m(A)}{2^{\left|A\right|}-1}e^{\frac{|A|-1}{\left|X\right|}}\right)$
Pan et al.’s Entropy [28]	$P_{Bel}\left(m\right)=-\sum_{A\subseteq X}^{}\frac{Bel(A)+Pl(A)}{2}\log_{2}\left(\frac{Pl(A)+Bel(A)}{2^{|A|-1}}\right)$
Cui et al.’s Entropy [29]	$E\left(m\right)=-\sum_{A\subseteq X}^{}m\left(A\right)\log_{2}\left(\frac{m(A)}{2^{\left|A\right|}-1}e^{\sum_{B\subseteq X,B\neq A}^{}\frac{\left|A\cap B\right|}{2^{\left|X\right|}-1}}\right)$

**Table 3:** The model of interval numbers.

Class	Alcohol	Malic Acid	Ash	OD	CI	Proline
1	[12.850,14.830]	[1.3500,4.0400]	[2.0400,3.2200]	[2.5100,4.0000]	[3.5200,8.9000]	[680,1680]
2	[11.030,13.860]	[0.7400,5.8000]	[1.3600,3.2300]	[1.5900,3.6900]	[1.2800,6.0000]	[278,985]
3	[12.200,14.340]	[1.2400,5.6500]	[2.1000,2.8600]	[1.2700,2.4700]	[3.8500,13.0000]	[415,880]
1,2	[12.850,13.860]	[1.3500,4.0400]	[2.0400,3.2200]	[2.5100,3.6900]	[3.5200,6.0000]	[680,985]
1,3	[12.850,14.340]	[1.3500,4.0400]	[2.1000,2.8600]	−	[3.8500,8.9000]	[680,880]
2,3	[12.200,13.860]	[1.2400,5.6500]	[2.1000,2.8600]	[1.5900,2.4700]	[3.8500,6.0000]	[415,880]
1,2,3	[12.850,13.860]	[1.3500,4.0400]	[2.1000,2.8600]	−	[3.8500,6.0000]	[680,880]

**Table 4:** BPAs based on Kang’s method.

Class	Alcohol	Malic Acid	Ash	OD	CI	Proline
m(1)	0.1699	0.1685	0.1416	0.2700	0.0967	0.0623
m(2)	0.0715	0.1095	0.0897	0.1732	0.2088	0.1700
m(3)	0.1244	0.1083	0.1568	0.1126	0.0562	0.1877
m(1,2)	0.1675	0.1685	0.1416	0.3168	0.1889	0.1187
m(1,3)	0.1860	0.1685	0.1568	0.0000	0.0939	0.1368
m(2,3)	0.1132	0.1083	0.1568	0.1273	0.1777	0.1877
m(1,2,3)	0.1675	0.1685	0.1568	0.0000	0.1777	0.1368

**Table 5:** Final BPA.

Class	Final BPA
m(1)	0.1515
m(2)	0.1371
m(3)	0.1243
m(1,2)	0.1837
m(1,3)	0.1237
m(2,3)	0.1452
m(1,2,3)	0.1345

**Table 6:** Fractional Deng entropies of BPAs in Table 4.

Attribute	Alcohol	Malic Acid	Ash	OD	CI	Proline
FDEn	2.2684	2.2658	2.2638	1.8801	2.2494	1.4378

**Table 7:** The weights of attributes based on FDEn.

Attribute	Alcohol	Malic Acid	Ash	OD	CI	Proline
Weight	0.1472	0.1473	0.1474	0.1775	0.1484	0.2322

**Table 8:** Final weighted BPA.

Class	Final Weighted BPA
m(1)	0.1474
m(2)	0.1411
m(3)	0.1293
m(1,2)	0.1822
m(1,3)	0.1210
m(2,3)	0.1483
m(1,2,3)	0.1307

**Table 9:** The recognition rate.

Non-Weighted Method	*q*	FDEn Method	FDEx Method
93.26%	0.5	94.38%	93.26%
	0.6	94.94%	93.26%
	1	94.38%	93.26%

## Data Availability

Publicly available datasets were analyzed in this study. This data can be found here: http://archive.ics.uci.edu/ml (accessed on: 18 April 2021).

## Abbreviations

The following abbreviations are used in this manuscript:

BPA	Basic probability assignment
CI	Color intensity
DST	Dempster–Shafer theory of evidence
FDEn	Fractional Deng Entropy
FDEx	Fractional Deng Extropy
FOD	Frame of discernment
OD	OD280/OD315 of diluted wines
PPT	Pignistic probability transformation
